# Silencing ROR1 and ROR2 inhibits invasion and adhesion in an organotypic model of ovarian cancer metastasis

**DOI:** 10.18632/oncotarget.22559

**Published:** 2017-11-20

**Authors:** Claire Henry, Neville Hacker, Caroline Ford

**Affiliations:** ^1^ Gynaecological Cancer Research Group, Lowy Cancer Research Centre and School of Women's and Children's Health, Faculty of Medicine, University of New South Wales, Sydney, Australia; ^2^ Gynaecological Cancer Centre, Royal Hospital for Women, Sydney, Australia

**Keywords:** ROR2, ROR1, epithelial ovarian cancer, omentum, metastasis

## Abstract

**OBJECTIVE:**

Elevated expression of the ROR1 and ROR2 Wnt receptors has been noted in both the tumour and stromal compartments of ovarian cancer patient tissue samples. *In vitro* studies have suggested these receptors play a role in ovarian cancer metastasis. However, these previous studies have utilised simple 2D *in vitro* models to investigate cancer cell growth and migration, which does not allow investigation of stromal involvement in Wnt driven metastasis.

**AIM:**

To investigate targeting ROR1 and ROR2 using a primary co-culture 3D model of epithelial ovarian cancer dissemination to the omentum.

**METHODS:**

Primary fibroblasts (NOF) and mesothelial (HPMC) cells were isolated from fresh samples of omentum collected from women with benign or non-metastatic conditions and cultured with collagen to produce a organotypic 3D model. Stable shRNA knockdown of ROR1, ROR2 and double ROR1/ROR2 in OVCAR4 cells were plated onto the 3D model to measure adhesion, or using a transwell to measure invasion. Gene expression changes in primary cells upon OVCAR4 interaction was evaluated using indirect transwell co-culture.

**RESULTS:**

Double knockdown of ROR1 and ROR2 strongly inhibited cell adhesion (p<0.05) and invasion (P<0.05) to the omentum model. ROR2 was up regulated in primary fibroblasts when cultured with OVCAR4 (P=0.05) and ectopic overexpression of ROR2 in NOFs inhibited cell proliferation (P<0.01) but increased cell migration.

**CONCLUSION:**

The combination of ROR1 and ROR2 signalling influences ovarian cancer dissemination to the omentum, however ROR2 may also play a role in stromal activation during metastasis. Therefore, targeting both ROR1 and ROR2 may be a powerful approach to treating ovarian cancer.

## INTRODUCTION

Approximately 60% of women with ovarian cancer will succumb to their disease within 5 years due to the lack of early detection tests, which means that patients present with advanced disease. Ovarian cancer has a different mode of spread from other solid haematogenous and lymphatic disseminating epithelial tumours. The generalised view of epithelial ovarian cancer (EOC) spread is that the primary cells shed into the peritoneal fluid and survive as tumour aggregates [1]. Extensive cell seeding throughout the peritoneal cavity is associated with ascites accumulation, which is often found in high grade serous ovarian cancer (HGSOC) [2]. The fatty apron which overhangs the small bowel (omentum) is often the first site of EOC metastasis and if the individual nodules coalesce, they form a large solid mass, referred to as an “omental cake” [2]. Omental involvement can cause obstructions to the large and small bowel, leading to painful abdominal cramping, vomiting or constipation [2].

The omentum consists of a protective barrier of mesothelial cells and underlying vascular adipose tissue. As the central regulator of peritoneal homeostasis, the omentum filters peritoneal fluid and provides a niche of growth factors and immune cells [3]. This specialised organ contains bands of adipose tissue mixed with stromal and immune cells. In particular, the omental vasculature contains ‘milky spots’, areas of glomerular-like capillary beds near the periphery of the tissue where macrophages and lymphocytes congregate, providing the majority of immunological defence in the peritoneal cavity [3]. Ovarian cancer cells preferentially colonise these areas on the omentum whilst adipocytes provide fatty acids as an energy source and adipokines to induce homing and invasion of ovarian cancer cells [4].

Dysregulated Wnt signalling has been implicated in a number of cancers and in recent years evidence to support the metastatic role of Wnt receptors ROR1 and ROR2 has built [5–8]. We previously demonstrated that silencing both ROR1 and ROR2 simultaneously significantly inhibited the ability of ovarian cancer cells to proliferate, migrate and invade *in vitro* [7]. These results were then supported in a chemoresistant cell line model [9], and by other studies using *in vivo* xenografts, where a ROR1 monoclonal inhibitor significantly decreased tumour burden [10, 11]. We recently identified an upregulation of ROR2 in stromal compartments of tissue samples from patients with metastatic disease [12]. Here we aimed to further investigate the role of these receptors in tumour and stroma, in the setting of omental metastasis using a patient derived organotypic model.

## RESULTS AND DISCUSSION

### ROR1 and ROR2 silencing inhibits ovarian cancer adhesion

**Figure 1:**
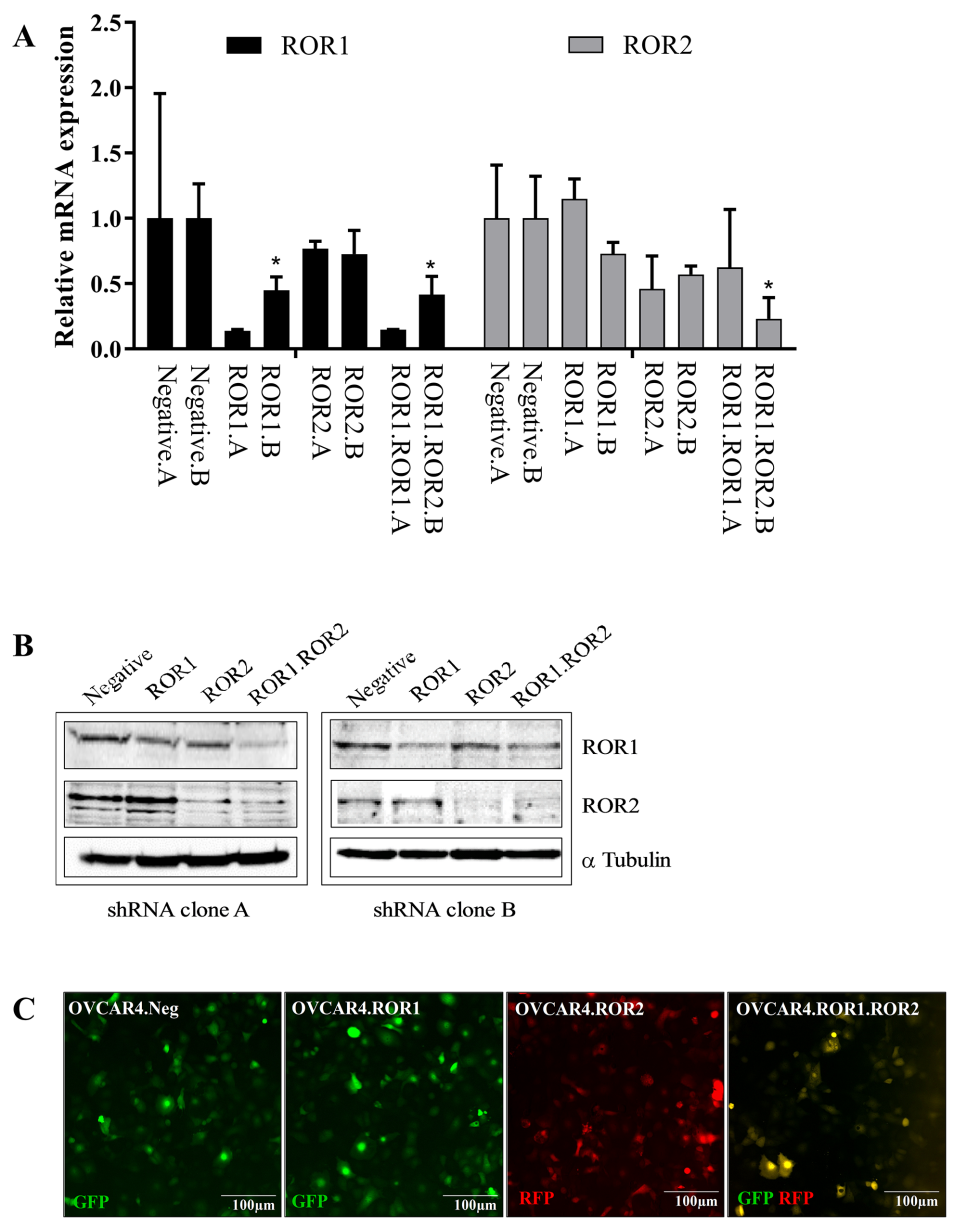
ROR1 and ROR2 knockdown in ovarian cancer cells **(A)** ROR1 expression is represented as black bars, ROR2 expression is represented as grey bars. Two separate clones (labelled A and B) are shown for each ROR shRNA. OVCAR4 shRNA clones A and B are paired for each condition (negative control, ROR1 siRNA, ROR2 siRNA and ROR1. ROR2 siRNA). Significant p-values represent comparison to corresponding clone negative control. ROR1 expression (Black bars) is decreased at the mRNA level following shRNA induced knockdown in OVCAR4, in clones A and B. No effect is seen on ROR1 levels in ROR2.shRNA cells. ROR2 (grey bars) is decreased at the mRNA level following shRNA induced knockdown in OVCAR4, in clones A and B. Significant p-values represent comparison to corresponding negative control. No effect is seen on ROR2 levels in ROR1.shRNA cells. qRT-PCR was performed in triplicate and normalised to three different housekeeping genes (SDHA, HSPCB, RPL13A). Results represent an average of four experiments. Error bars represent the s.d of the mean. **(B)** Representative Western blots reflect protein expression of ROR1 and ROR2 knockdown in shRNA treated OVCAR4. Left panel: clone A, right panel: clone B. In each panel, top row ROR1, middle row ROR2, bottom row α-tubulin (loading control). **(C)** Representative fluorescence images of Negative shRNA (GFP expressing), ROR1 shRNA (GFP expressing), ROR2 shRNA (RFP expressing) and double knockdown ROR1 and ROR2 shRNA (GFP and RFP expressing) OVCAR4 cells.

The ovarian cancer cell line OVCAR4 was used in this study as it has been previously identified as a suitable cell line to represent HGSOC [13] and forms papillary structured tumours in xenografts [14]. OVCAR4 lines were stably transduced with shRNA targeting ROR1, ROR2, both ROR1 and ROR2 or a non-targeting control. Two separate shRNA clones (clone A and clone B) were developed and successful knockdown of RORs was confirmed through RNA expression analysis and Western blotting (Figure 1A-1B). ROR2 shRNA transduction of OVCAR4 did not result in a large decrease in ROR2 RNA expression due to the already low base level of ROR2, however successful reduction was observed at the protein level (Figure 1B). shRNA constructs contained GFP or RFP genes which also confirmed integration of shRNA constructs into OVCAR4 cells (Figure 1C).

**Figure 2:**
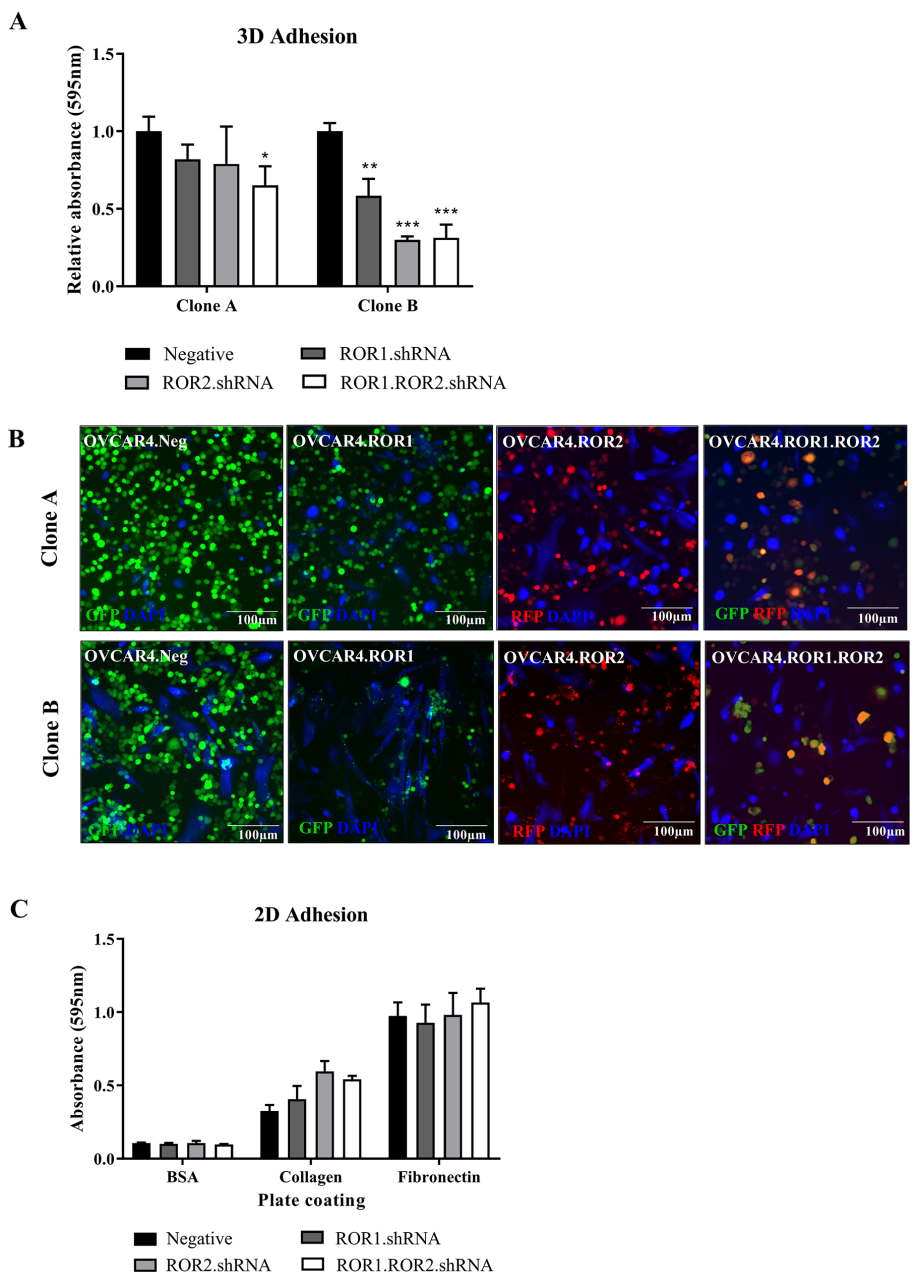
ROR1 and ROR2 silencing decreases OVCAR4 adhesion to omentum model **(A)** ROR1 and Double ROR1/ROR2 shRNA OVCAR4 adhere significantly less to 3D model when compared to negative control in both clone A (left set) and clone B (right set) (^*^P<0.05, ^**^<0.01, ^***^<0.001, n=3). Results represent an average of three experiments. Error bars represent the s.d of the mean. **(B)** Representative fluorescent images of adhesion assay for clone A (top panel) and clone B (bottom panel). NOFs treated with cell tracker CMAC are shown in blue (DAPI). **(C)** 2D adhesion assay using clone B OVCAR4 shRNA cells. No significant change between ROR1, ROR2 or ROR1 and ROR2 knockdown. Results represent an average of three experiments. Error bars represent the s.d of the mean.

Adhesion to the omentum is an integral step in ovarian cancer metastasis. Using a 3D organotypic model comprised of stromal cells and collagen, we analysed the adhesive capacity of ROR1, ROR2 and double ROR1/ROR2 depleted OVCAR4 cells. Whilst both shRNA clones exhibited a loss of adhesion to the model particularly in the double ROR1 and ROR2 knockdown (P<0.05 Clone A, P<0.001 clone B, Figure 2A), it was the clone B that showed the most significant reduction (Figure 2A-2B). As this was the first time an effect on adhesion in ROR silenced ovarian cancer cells has been observed, a previously described simple 2D adhesion assay (using collagen and fibronectin coated plates) was conducted using clone B set of OVCAR4 knockdown [7]. There were no significant changes to OVCAR4 adherence to collagen or fibronectin using this protocol (Figure 2C). These differing results highlight the importance of using complex organotypic 3D models to better investigate receptor function *in vitro*. Analysis of adhesion related genes in both clone A and B revealed mixed RNA expression profiles (Supplementary Figure 2A), though this is based on OVCAR4 cells growing alone and without contact with a microenvironment. It would be important to continue the investigation of ROR receptors in adhesion after contact with a 3D environment to understand the signalling dynamics involved. Our study found that depleting ovarian cancer cells of both ROR1 and ROR2 decreased adhesion capacity and was reflected in mixed mRNA expression profiles of adhesion associated genes. The profile of set B at the time of analysis may reflect the stronger results seen in Figure 2. This may be due to the strength of knockdown in these samples, or the length of time each set was cultured before the adhesion assay; the effect of long term culturing with ROR knockdown is unknown. Of note, DDR1 is a receptor tyrosine kinase involved in cell-microenvironmental communication activated by collagen I [15] and associated with worse prognosis and late stage EOC [16]. Collagen induced DDR1 phosphorylation has been linked to WNT5A and Src tyrosine kinase activity [17, 18]. Interestingly, both ROR1 and ROR2 have been shown to phosphorylate Src in different contexts [19, 20] and are both receptors activated by the WNT5A ligand. The opposing expression patterns in set A compared to set B of this molecule was observed in our ROR silenced cells, however it would be important to extend analysis into DDR2 expression using both qRT-PCR and immunocytochemistry to view localisation of the proteins. In each clone, MMP7 and CD44 increased with ROR1 and ROR2 knockdown, respectively. MMP7 in fact increases ovarian cancer invasion [21–23] and overexpression of CD44, a cancer stem cell marker promotes development of metastasis and recurrence [24–26]. Our functional results do not reflect these mRNA expression patterns and therefore warrants further investigation and analysis of pathway and protein interactions. Other MMPs previously implicated in ovarian cancer progression such as MMP2, MMP8 and MMP14 [27–30] were also analysed however OVCAR4 expressed low to undetectable mRNA levels of these (unpublished data).

### ROR1 and ROR2 silencing inhibits cell invasion

**Figure 3:**
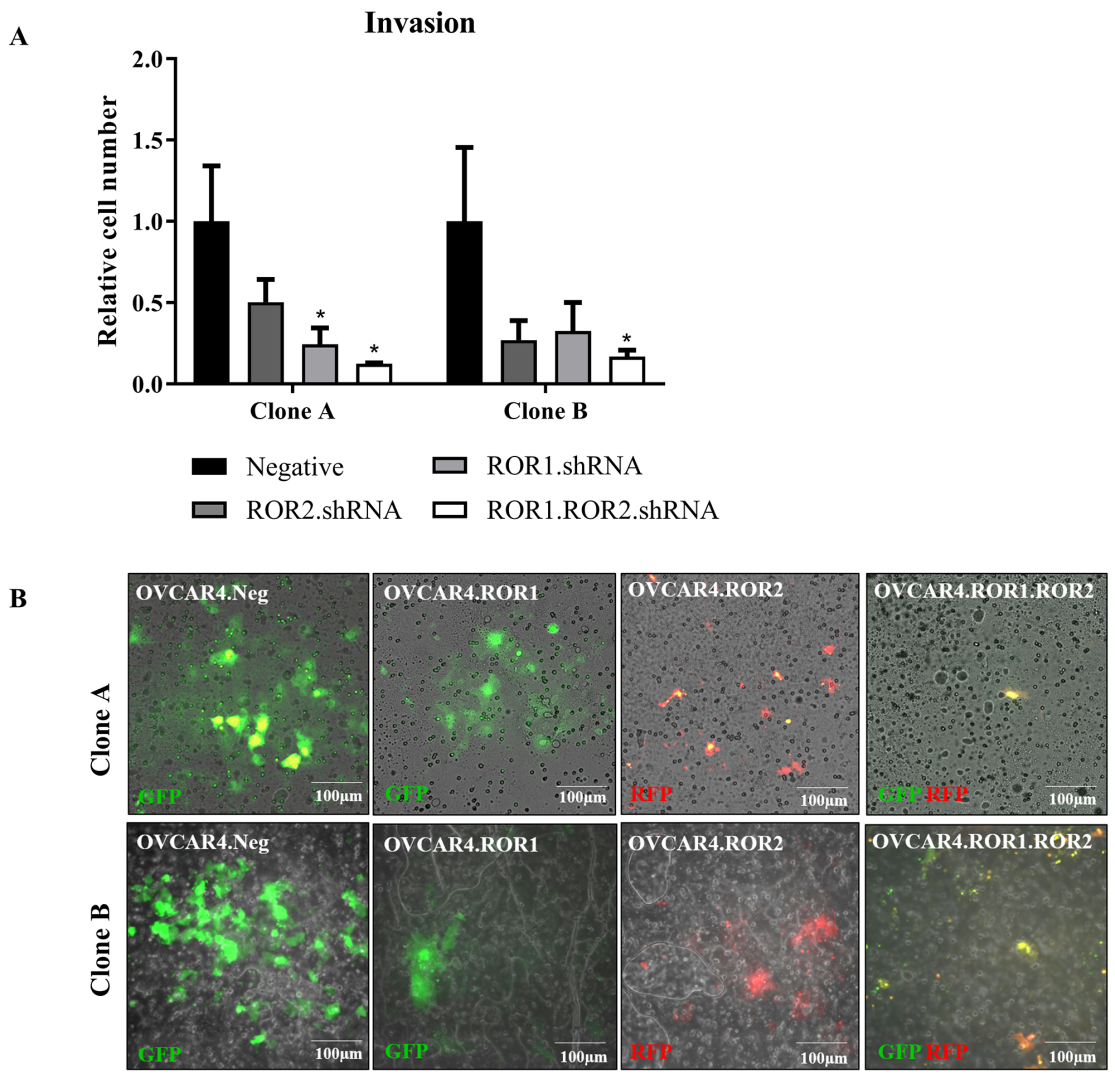
ROR1 and ROR2 silencing decreases OVCAR4 invasion into omentum model **(A)** Graph represents average number of cells invaded through membrane for both clone A and clone B after 48 hours. ROR1 and ROR2 shRNA cells had decreased invasive capacity however double ROR1 and ROR2 shRNA was most significant (P<0.05, n=3). Results represent an average of three experiments. Error bars represent the s.d of the mean **(B)** Representative images of fluorescently tagged OVCAR4 cells invaded through membrane. Top panel: clone A, bottom panel: clone B.

We have previously shown that double ROR1 and ROR2 knockdown decreases ovarian cancer cell invasion into a matrigel layer [7, 9]. However, this has not been shown in a complex organotypic model with additional co-cultured stromal cells, which is important as ovarian cancer cells alter protein expression, proliferation and chemosensitivity in a 3D microenvironment [31]. Therefore, we wished to investigate and validate our previous results in a new more complex and clinically relevant setting. We observed that whilst ROR1 and ROR2 individual shRNA clones had decreased capacity to invade through the co-culture, it was the double ROR1 and ROR2 depleted OVCAR4 that had significantly impaired invasion in both shRNA clones (Figure 3A-3B). This aligns with our other previous reports in a number of different ovarian cancer cell lines [7, 9].

This study confirmed the role of ROR1 and ROR2 in omental invasion. Directions for future studies should continue to investigate ROR1 and ROR2 in ovarian cancer metastasis through mouse models and assessment of tumour nodules on omentum and peritoneum as previously described [4]. The Cysteine-Rich Domain of the ROR receptor structure is critical for Wnt binding and ROR activation, whilst the Kringle Domain is responsible for ROR heterodimerisation [32]. Therefore, one potential proposition based on this study is to create, and trial in mouse models, a small molecule inhibitor able to bind and inhibit both domains to inactive all ROR downstream signalling.

### ROR2 plays a role in ovarian cancer stroma

**Figure 4:**
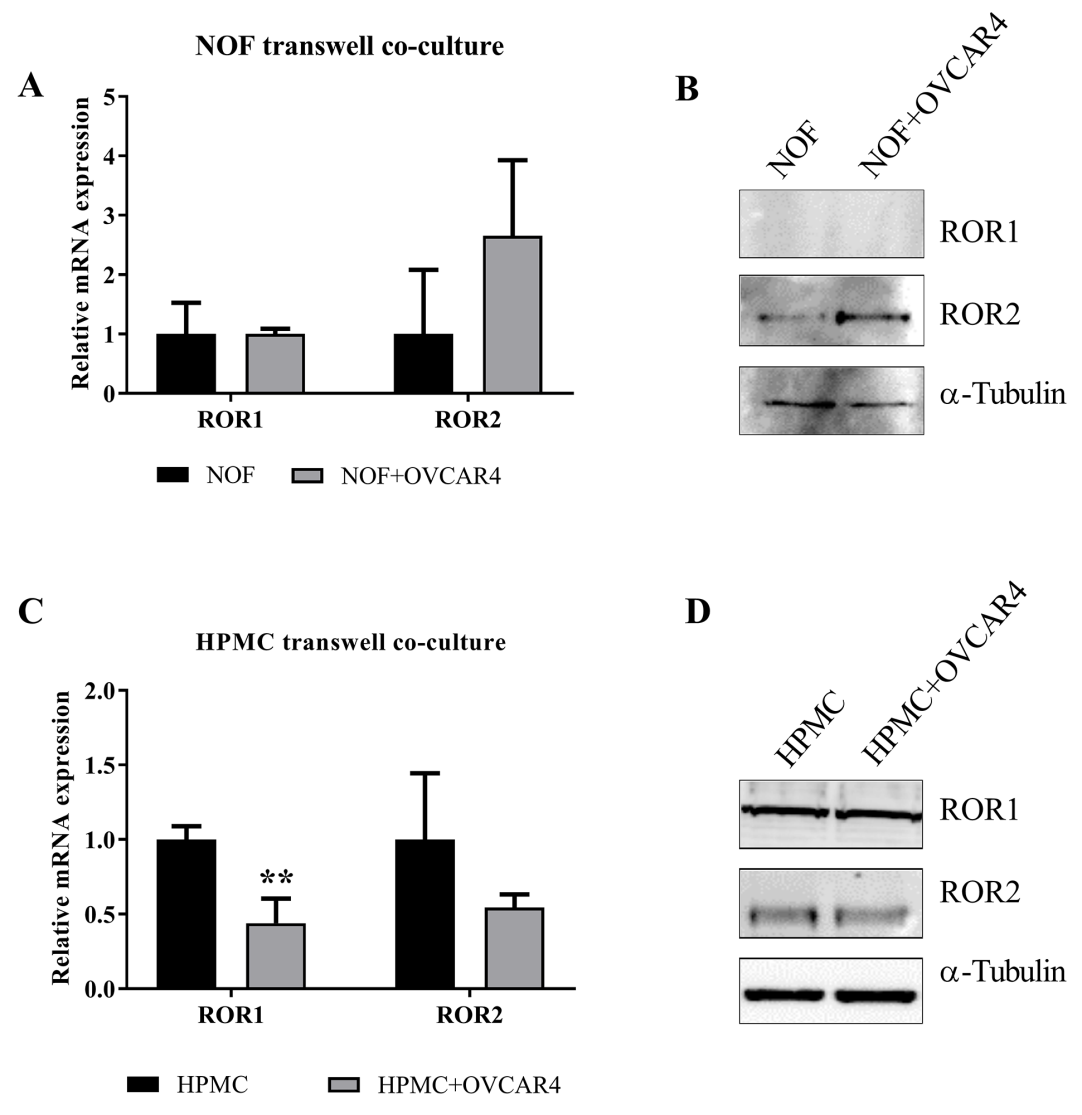
Indirect co culture of HPMC and NOFs with OVCAR4 affect stromal ROR expression **(A)** mRNA expression of ROR1 and ROR2 in NOF (Black bar) and NOF cultured with OVCAR4 (Grey bar). A significant increase in ROR2 mRNA was found when NOF was cultured with OVCAR4 (p=0.05, n=3) and no change in ROR1 mRNA observed. qRT-PCR was performed in triplicate and normalised to three different housekeeping genes (SDHA, HSPCB, RPL13A). Results represent an average of three experiments. Error bars represent the s.d of the mean. **(B)** Representative Western blot analysis of protein expression of ROR1 (top panel), ROR2 (middle panel) and loading control α-tubulin (bottom panel) shows an increase in ROR2 in NOF cultured with OVCAR4 cells reflecting mRNA expression, whilst no expression of ROR1 protein is detected. **(C)** mRNA expression of ROR1 and ROR2 in HPMC (Black bar) and HPMC cultured with OVCAR4 (Grey bar). A significant decrease in ROR1 mRNA was found when HPMC was cultured with OVCAR4 (p<0.01, n=3). qRT-PCR was performed in triplicate and normalised to three different housekeeping genes (SDHA, HSPCB, RPL13A). Results represent an average of three experiments. Error bars represent the s.d of the mean. **(D)** Representative Western blot analysis of protein expression of ROR1 (top panel), ROR2 (middle panel) and loading control α-tubulin (bottom panel) in HPMC cultured with OVCAR4.

We previously found ROR2, but not ROR1 upregulation in ovarian cancer associated stroma, in particular in metastatic samples of matched patient study cases [12]. ROR2 expression in stroma has otherwise only been reported in pancreatic cancer [33]. Therefore, we conducted a preliminary assessment to see if this was reproducible *in vitro*. A transwell was used to separate OVCAR4 ROR2 positive (negative control shRNA) cells from NOFs in a co-culture setting over 48 hours. We observed that NOFs cultured with OVCAR4 had a significant increase in ROR2 mRNA, but not ROR1 mRNA, when compared to NOFs cultured alone (Figure 4A, P=0.05). Stromal cells showed very low protein expression of both ROR1 and ROR2 at base level, however only ROR2 was increased in NOFs cultured with OVCAR4 (Figure 4B). In addition, HPMCs which line the omentum and come in first contact with ovarian cancer cells during metastasis were also subject to OVCAR4 co culture to assess if these cells responded in a similar fashion. In fact, HPMCs exhibited a decrease in ROR1 and ROR2 at the mRNA (Figure 4C, ROR1 P<0.01) level however no change was observed in protein expression of either receptor (Figure 4D) when cultured with OVCAR4.

The WNT5A-ROR2-JUN pathway has been previously implicated in fibrosis related pathologies such as keloid scars and scleroderma [34, 35]. There is some evidence that the role of the inflammatory peritoneum acts as a facilitator of the pro tumour microenvironment, which can increase the malignant potential of ovarian cancer cells [36]. Ovarian cancer patients with a ‘fibrosis’ gene signature have been shown to have partial debulking and incomplete response to chemotherapy [37].

**Figure 5:**
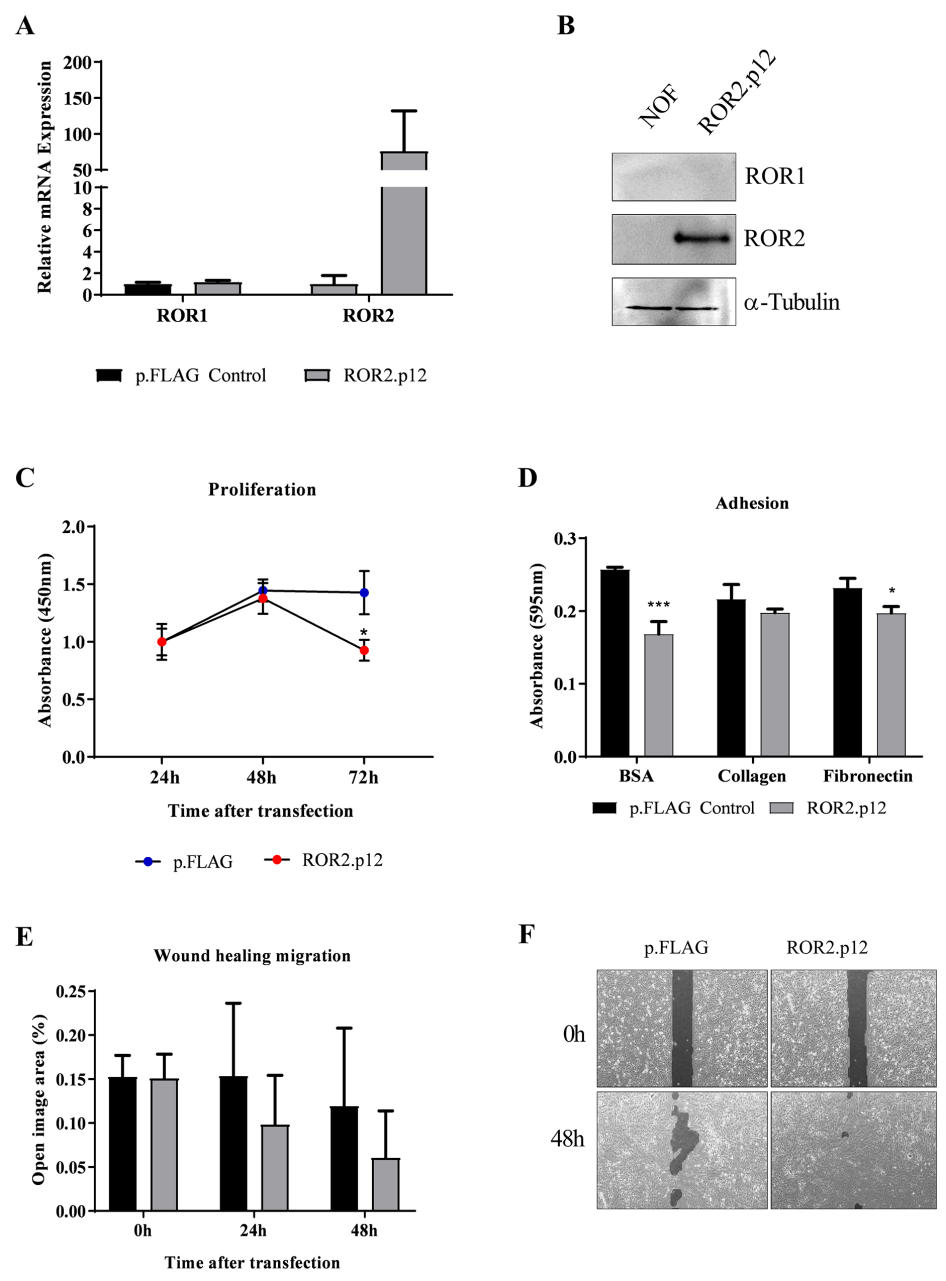
ROR2 overexpression in NOFs increases migration **(A)** Ectopic overexpression of ROR2 in NOFs increases mRNA levels over 100-fold, with no change to ROR1. qRT-PCR was performed in triplicate and normalised to three different housekeeping genes (SDHA, HSPCB, RPL13A). Error bars represent the s.d of the mean, repeated three times. **(B)** Representative Western blot analysis of protein expression of ROR2 (top panel), ROR1 (middle panel) and loading control α-tubulin (bottom panel) shows successful transfection of ROR2 plasmid in NOFs. **(C)** Significant loss of proliferation in NOFs is seen after 48 hours of initial ROR2 overexpression (Red circles, P<0.05, n=3). **(D)** 2D adhesion to BSA control, Collagen and fibronectin after ROR2 ectopic expression in NOFs (grey bars). Significant decrease in adhesion after ROR2 overexpression is observed (P<0.05, n=3). **(E)** NOF wound healing capacity is increased, as indicated by a decrease in ‘open image area’, after ectopic ROR2 overexpression (grey bar). **(F)** Representative images of ‘open image area’ (dark grey shading) measured in wound healing assay.

Because of the novel findings, we conducted preliminary ROR2 overexpression in NOF cells to investigate the role of ROR2 in the ovarian cancer microenvironment, using transient overexpression of a previously developed ROR2 plasmid [7]. ROR2 overexpression was successfully achieved for the first time in these primary cells, and had no effect on levels of ROR1, as measured at both the RNA and protein expression levels (Figure 5A-5B). Interestingly, we observed a decrease in cell proliferation after ectopic ROR2 expression compared to the empty vector control (Figure 5C), yet an increase in wound healing migration (Figure 5E-5F). We observed that NOF cells transfected with empty p.FLAG control tended to proliferate on top of each other, rather than spreading into the wound area. When applied to the 2D adhesion assay, NOFs had no preference to adhere to collagen or fibronectin over the BSA control (Figure 5D), in contrast to the OVCAR4 cells (Figure 2C). Interestingly, ROR2 overexpression decreased NOF adherence to the BSA control (P<0.001 Figure 5G) and fibronectin (P<0.05 Figure 5D). As the Wnt5a-ROR2-Jun pathway has been related to fibrosis pathologies, we performed a preliminary analysis of genes associated with collagen production and fibrosis. Ectopic ROR2 overexpression increased the mRNA expression of COL1A2 and SMA in NOFs (Supplementary Figure 2B), indicating a possible role for ROR2 in cancer associated stromal activation in the context of ovarian cancer dissemination to the omentum.

## MATERIALS AND METHODS

### Omentum collection and ethics

Ethics approval was obtained for the collection of patient omentum through the South Eastern Sydney Local Health District Human Research Ethics Committee (SESLHD HREC, Ethics no. 16/108). Site specific approval for the collection at Royal Hospital for Women, Randwick, was also obtained (Ethics no. 16/G/154). Healthy omentum biopsies approximately 1cm^3^ in size were donated by patients who were undergoing surgery for benign or low grade gynaecological malignancies. All biopsies used for subsequent assays were confirmed to be free of tumour cells by a pathologist.

### Isolation and culture of primary untransformed stromal cells

Processing of healthy omentum samples is summarised in Supplementary Figure 1 and is based on previously described methods [38].

One hour before omentum preparation, flasks were coated with 10μg/ml fibronectin in Dulbecco's Phosphate Buffered Saline (PBS) to aid in the attachment and growth of Human Primary Mesothelial Cells (HPMC). The omentum was collected, washed and scraped twice. Harvested PBS containing mesothelial cells were centrifuged and resuspended in full growth media (DMEM with 10% FBS, 1% MEM non essential amino acids and 1% pen/strep). HPMCs were grown at 37°C and 5% CO_2_ in a humidified environment.

Remaining omentum from HPMC isolation was digested to isolate Normal Omentum Fibroblasts (NOFs). Omentum was minced and digested in 10X Collagenase type I solution (Sigma-Aldrich, Castle Hill NSW Australia) in TESCA buffer (50 mM TES, 0.36 mM Calcium chloride solution, pH 7.4) at 37°C on a shaker at 200 rpm for approximately 6 hours or until no solid tissue remained. The solution was then centrifuged and the pellet was resuspended in full growth media (see HPMC).

Phenotype and cell markers were assessed to confirm isolation of HPMCs and NOFs. HPMCs exhibited a cuboidal shape whereas NOFs were flat, elongated cells (Supplementary Figure 1). Immunocytochemistry confirmed expression of cytokeratin 8 (CK8) and vimentin in the HPMCs and NOFs. HPMCs but not NOFs express cytokeratin 8 whereas they both express vimentin (Supplementary Figure 1). Briefly, cells were seeded onto sterilised coverslips in individual 60mm culture dishes at a concentration of 5×10^5^cells/ml. After 24 hours, cover slips were washed with PBS and fixed with 4% paraformaldehyde. Cells were then permeabilised with 0.5% Triton X-100 then 1% Hydrogen Peroxide. Goat serum (10%) in PBS was used for blocking and cover slips were subsequently incubated with primary antibodies overnight at 4°C. Primary antibodies were used at a 1:500 dilution (anti-Vimentin, Cell signalling #5741 and anti-CK8, Abcam #58230). The following day, cover slips were washed in PBS and incubated with HRP-coupled goat anti-Rabbit IgG (1:100, DAKO, Agilent pathology solutions, Mulgrave VIC Australia). Cells were then visualised using DAB solutions kit (ab64238, Abcam, Melbourne VIC Australia) and counterstained with haematoxylin.

### NOF transwell co culture

NOF and OVCAR4 cells were co cultured using transwell inserts (Corning Life Sciences, Tewksbury, MA, USA) for separation. 1×10^3^ NOF cells were plated in 24 well plates and left to adhere for 24 hours. OVCAR4 cells were then added to transwells sitting above NOFs at the same concentration and left to grow for 48 hours at 37°C. Transwells were subsequently removed, NOFs were washed with PBS and harvested for RNA and protein analysis.

### ROR2 overexpression in NOF cells

ROR2 pFLAG and negative control empty vector pFLAG plasmids as previously described [9] were used for ROR2 overexpression in NOF cells. Briefly, 1×10^6^ cells/ml were seeded in 6 well plates and serum starved overnight. Cells were transfected with 2500ng of either empty p.FLAG plasmid or ROR2 plasmid using Lipofectamine 3000 and p3000 reagent as per manufacturers instructions (Invitrogen, Carlsbad CA, USA).

### Proliferation assay

Proliferation of NOFs was measured using CCK8 as previously described [9] and as per manufacturers instructions (Dojindo, Rockville, MD, USA). Briefly, 6-8 hours after transfection, 100 μl of cells were seeded in triplicate into a 96-well plate at a concentration of 4×10^4^ cells/ml. Proliferation was measured 24, 48 and 72 hours after initial transfection using an absorbance reading of 450 nm using the SpectraMax 190 Microplate reader (Molecular Devices, Sunnydale, CA, USA). Each assay was repeated in triplicate.

### Wound healing assay

Wound healing was measured using IBIDI insert plates as previously described [7]. Cells were plated onto IBIDI plates at a concentration of 1×10^5^ cells/ml. Culture inserts were removed after cells had adhered (24 hours). Photographs of IBIDI plates were taken at 0, 24 and 48 hour time points beginning from insert removal using a Leica DMIL microscope (Leica Microsystems, North Ryde, NSW, Australia). Wound healing was then analysed using TScratch software (ETH Zurich, Zurich, Switzerland) [39]. Each assay was repeated in triplicate.

### 2D adhesion assay

2D adhesion to collagen or fibronectin was performed as previously described [7]. Briefly, 24 well plates were coated with collagen (10ug/ml), fibronectin (5ug/ml) or control BSA (3%) for 24 hours before seeding with NOF or OVCAR4 cells (5×10^5^ cells/ml) for 3 hours. Plates were subsequently washed, fixed with 96% ethanol and stained with 1% crystal violet. Adhered cells were lysed in 50% Acetic and solution was read on SpectraMax 190 Microplate reader (Molecular Devices, Sunnydale, CA, USA) at an absorbance of 595mm. An increase in absorbance relates to an increase in cell adhesion and each assay was repeated in triplicate.

### OVCAR4 shRNA stable knockdown

Stable knockdown in OVCAR4 cells were created using a lentiviral transduction system (GTRC approval NLRD 16-19). Mission shRNA ready-made lentiviral particles were purchased from Sigma (Sigma-Aldrich, Castle Hill NSW Australia). Previously validated, custom lentiviral pLKO.1-CMV vectors were used as follows: ROR1 (pLKO.1-Neo-CMV-tGFP, #TRCN038784R12024), ROR2 (pLKO.1-puro-CMV-TagRFP, #TRCN038784R11492) and non targeting (pLKO.1-Neo-CMV-tGFP, #SHC004V).

OVCAR4 cells were transduced in 24 well plates by ready-made lentiviral particles at a multiplicity of infection (MOI) rate of 1. Polybrene (hexadimethrine bromide) was used at a concentration of 8μg/ml to aid transduction. After transduction, cells were washed extensively and left for 48 hours. Positive GFP or RFP fluorescence indicated successful transduction. Cells with integrated vectors were then selected for using either puromycin (8μg/ml) or G148 (800μg/ml). Fluorescence activated cell sorting (FACs) was used to select high expressing GFP or RFP cells, provided by the Biomedical Imaging Facility at UNSW Australia. Double knockdown clones were achieved by repeated transduction of ROR1-shRNA-OVCAR4 with lentiviral particles containing ROR2 shRNA.

### RNA analysis

mRNA extraction and cDNA synthesis was carried out using the RNeasy Mini-Kit (Qiagen, Valencia, CA, USA) following the manufacturer's instructions [9]. After DNase treatment, 1 μg of RNA was converted to cDNA using the Quantitect RT kit (Qiagen, Valencia, CA, USA). qPCR was performed in triplicate on a Stratagene MxPro 3005P machine using 25 ng of cDNA, 100 nM of primers and 12.5 μL SYBRGreen Dye (Qiagen) in each reaction. Expression values were normalised against house-keeping genes Succinate Dehydrogenase Complex Subunit A (SDHA), 90 kDA Heat Shock Protein 1 Beta (HSPCB) and 60S Ribosomal ProteinL13a (RPL13A) using the Vandesompele normalisation method [7]. Primer sequences are listed in Supplementary Table 1.

### Western blots

Western blots were performed as previously described [9]. Protein lysates were separated on an 8 % sodium dodecyl sulfate (SDS)-polyacrylamide gel. Primary antibodies used were anti-ROR1 (polyclonal Ab #AF2000, R&D Systems, Minneapolis, MN, USA) and anti-ROR2 (#34045, QED Bioscience, San Diego, CA, USA). Membranes were visualized using chemiluminescence ECL solutions and quantified using the ImageQuant LAS4000 (GE Healthcare Life Sciences, Parramatta, NSW, Australia).

### Organotypic 3D co-cultures

The organotypic culture was assembled as previously described [38]. NOFs (2-4×10^3^/100μl) were mixed with collagen (rat tail collagen type I, Gibco, Carlsbad, CA, USA) at a concentration of 0.5μg/100μl and plated onto a black, clear bottomed 96 well plate. The cells were left to adhere for 4 hours.

HPMCs were then released from the flask and plated on top of the NOF/Collagen mix at a concentration of 1-2×10^4^ cells/50μl. Cells were then left to incubate overnight before subsequent assays were performed.

Stable ROR1, ROR2 and double ROR knockdown OVCAR4 cells were utilised in all following assays. Cell tracker blue CMAC dye (Thermofisher Scientific, MA, USA) was used to stain stromal cells for fluorescent imaging and has an excitation/emission spectra of 353/466nm, well separated from GFP and RFP that are used to confirm shRNA transfection of ROR1 and ROR2 shRNA constructs in OVCAR4 cells respectively. Briefly, before NOF or HPMC plating, adhered cells were treated with Cell Tracker CMAC dye (Thermofisher Scientific, MA, USA) for 30 minutes at a concentration of 20μM as per manufacturer's instructions.

### 3D adhesion assay

Following co-culture overnight incubation, the plates were inverted to remove spent media without disrupting the layers. Ovarian cancer cells were diluted to a concentration of 5×10^4^cells/100μl in serum free media, and added to each well of the co-culture. The plate was then incubated at 37°C, 5% CO_2_ for 3 hours. After incubation, the plate was inverted again to remove media and non-adherent cells. The plate was gently washed with PBS and inverted (twice) to remove all remaining non-adherent cells. Cells were then fixed in 4% Paraformaldehyde and representative fluorescent images were taken on a tissue culture Zeiss Axio Microscope (Carl Zeiss, Jena, Germany). Wells were then stained using 1% Crystal Violet for thirty minutes. Cultures were lysed using 50% Acetic Acid and absorbance was read at 595nm. An increase in absorbance was equal to an increase in cell attachment. Each assay was repeated in triplicate.

### Invasion assay

Invasion of modulated OVCAR4 cells through the 3D culture was measured using transwells as previously described [38]. Before creating the culture inside the transwell assay, 7.5μg collagen I in 200μl PBS was added to each insert and incubated overnight.

Following incubation, PBS was carefully removed and HMPC/NOF culture was plated into inserts as described above. Prepared stable knockdown OVCAR4 cells were then added above the co-culture in serum free media at 1×10^5^cells/ml, and full growth media was added below the transwell. Transwells were then incubated for 48 hours at 37°C, 5% CO_2_.

Transwell membranes were washed, fixed with 4% paraformaldehyde and mounted on slides, where the number of invaded fluorescent cells were counted using Image J (Java Software) and averaged between four representative images of the membrane. Each assay was repeated in triplicate.

### Statistics

All statistical analysis were performed as previously described [9]. Results are expressed as the mean ± standard deviation (SD). An F test was first performed to determine unequal or equal data variance before significance was determined using a student's *t* test type 2. *T* test values below P < 0.05 were considered statistically significant. ^*^P < 0.05, ^**^P < 0.01, ^***^P < 0.001.

## CONCLUSIONS

This study was the first to investigate the metastatic potential of ROR depleted ovarian cancer cells using a unique 3D organotypic model. We have confirmed previous findings that ROR1 and ROR2 have a synergistic role in ovarian cancer invasion, and have presented new evidence that they are also important in adherence to omentum, the critical first step in ovarian cancer metastasis. We have also uncovered a potential pro-metastatic role for ROR2 upregulation in the omental stroma, which needs to be robustly investigated using 3D models as outlined. Combination therapy against both ROR receptors may provide a powerful approach to targeting two facets of metastasis; the tumour and the reactive stroma, thereby reducing inoperable widespread disease common in ovarian cancer patients.

## SUPPLEMENTARY MATERIALS FIGURES AND TABLES



## Abbreviations

ROR: Receptor Tyrosine Kinase like Orphan Receptor
EMT: Epithelial to Mesenchymal Transition
EOC: Epithelial Ovarian Cancer
HGSOC: High Grade Serous Ovarian Cancer
HPMC: Human Primary Mesothelial Cells
NOF: Normal Omentum Fibroblast
