# Supplementary material for: Silencing ROR1 and ROR2 inhibits invasion and adhesion in an organotypic model of ovarian cancer metastasis
Source: Oncotarget. 2017 Nov 20;8(68):112727–38. doi: 10.18632/oncotarget.22559 (PMC5762545; doi:10.18632/oncotarget.22559)
Supplement: Supplementary file 1 [file oncotarget-08-112727-s001.pdf]

Silencing ROR1 and ROR2 inhibits invasion and adhesion in an organotypic model of ovarian cancer metastasis

SUPPLEMENTARY MATERIALS

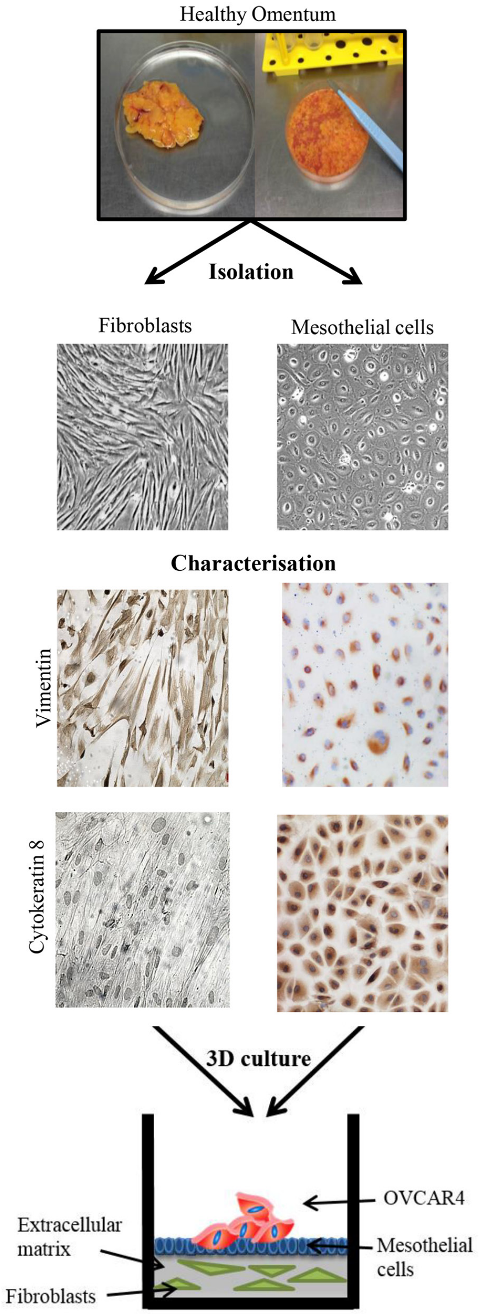

Supplementary Figure 1: Flow chart of omentum processing. Omentum is washed and scrapped to isolate HPMCs and further digested with collagenase to digest NOFs. HPMCs exhibit a cuboidal, round shape whereas NOFs exhibit long, elongated shapes. Confirming immunocytochemistry shows HPMCs express vimentin and cytokeratin 8 whereas NOFs only express vimentin. NOFs (green triangles) mixed with collagen (grey) are plated first in the 3D culture, followed by HPMCs (blue) to create a 3D organotypic model of omentum metastasis with cancer cells (red) added on top.

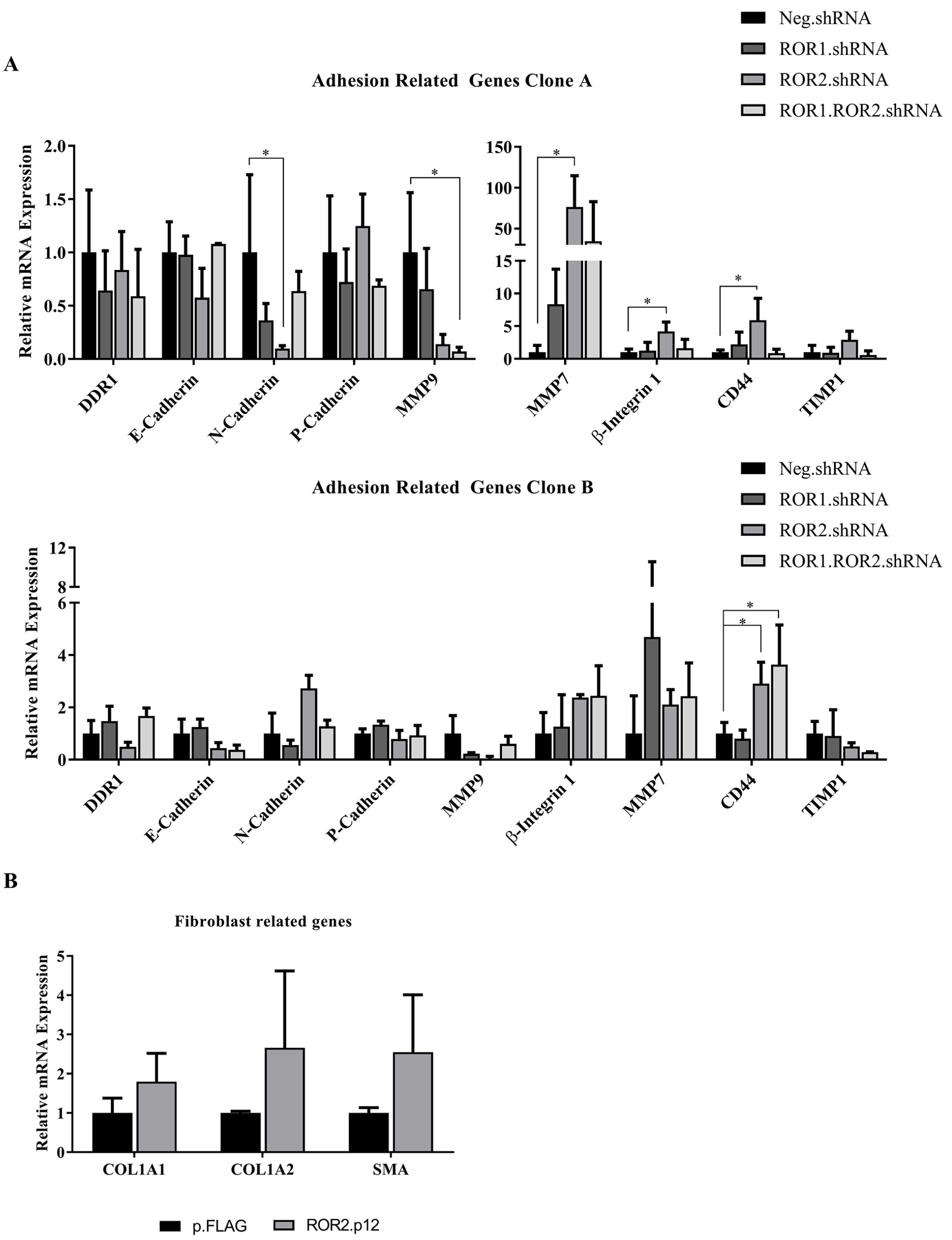

Supplementary Figure 2: RNA analysis of associated genes. (A) mRNA expression relative to negative control OVCAR4 cells of adhesion associated genes in shRNA clone A (top two panels) and shRNA clone B (bottom panel). qRT-PCR was performed in triplicate and normalised to three different housekeeping genes (SDHA, HSPCB, RPL13A). Results represent an average of three experiments. Error bars represent the s.d of the mean. **(B)** mRNA expression of Smooth Muscle Actin (SMA), Collagen 1 A1 (COL1A1) and Collagen 1 A2 (COL1A2) in ROR2 overexpressing NOFs, relative to p.FLAG control. qRT-PCR was performed in triplicate and normalised to three different housekeeping genes (SDHA, HSPCB, RPL13A). Results represent an average of three experiments. Error bars represent the s.d of the mean.

Supplementary Table 1: Primer sequences used for qRT-PCR

Gene	Forward primer 5'-3'	Reverse primer 5'-3'
**ROR1**	CAACAAGAAGCCTCCCTAATGG	CCTGAGTGACGGCACCTAGAA
**ROR2**	CGACTGCGAATCCAGGACC	GGCAGAACCCATCCTCGTG
**DDR1**	AATCGCAGACTTTGGCATGAG	CGTGAACTTCCCCATGAGGAT
**CDH1**	AGGCCAAGCAGCAGTACATT	ATTCACATCCAGCACATCCA
**CDH2**	ACAGTGGCCACCTACAAACG	CCGAGATGGGGTTGATAATG
**CDH3**	CACGACCTCATGTTCACCAT	CTCAGGGACTTTTTCCCGGT
**INTEGRINβ1**	GGATTCTCCAGAAGGTGGTT	GGTAAAACAATGCCACCAAG
**MMP9**	ACTGAGAGGCTCCGAGAAATG	GAACCCCGCATCTTGGCTT
**MMP7**	GATGAGGATGAACGCTGGAC	GCTAAATGGAGTGGAGGAACAG
**CD44**	CCTCTTGGCCTTGGCTTTG	TCCATTGCCACTGTTGATCA
**TIMP1**	GGGGCTTCACCAAGACCTAC	GGAAGCCCTTTTCAGAGCCT
**COL1A1**	ACGAAGACATCCCACCAATCAC	CGTTGTCGCAGACGCAGAT
**COL1A2**	GAGGGCAACAGCAGGTTCACTTA	TCAGCACCACCGATGTCCAA
**SMA**	CCGACCGAATGCAGAAGGA	ACAGAGTATTTGCGCTCCGAA
**RPL13A**	CCTGGAGGAGAAGAGGAAAGAGA	TTGAGGACCTCTGTGTATTTGTCAA
**SDHA**	TGGGAACAAGAGGGCATCTG	CCACCACTGCATCAAATTCATG
**HSPCB**	TCTGGGTATCGGAAAGCAAGCC	GTGCACTTCCTCAGGCATCTTG
